# Risk Factors and Outcomes for Late Presentation for HIV-Positive Persons in Europe: Results from the Collaboration of Observational HIV Epidemiological Research Europe Study (COHERE)

**DOI:** 10.1371/journal.pmed.1001510

**Published:** 2013-09-03

**Authors:** Amanda Mocroft, Jens D. Lundgren, Miriam Lewis Sabin, Antonella d'Arminio Monforte, Norbert Brockmeyer, Jordi Casabona, Antonella Castagna, Dominique Costagliola, Francois Dabis, Stéphane De Wit, Gerd Fätkenheuer, Hansjakob Furrer, Anne M. Johnson, Marios K. Lazanas, Catherine Leport, Santiago Moreno, Niels Obel, Frank A. Post, Joanne Reekie, Peter Reiss, Caroline Sabin, Adriane Skaletz-Rorowski, Ignacio Suarez-Lozano, Carlo Torti, Josiane Warszawski, Robert Zangerle, Céline Fabre-Colin, Jesper Kjaer, Genevieve Chene, Jesper Grarup, Ole Kirk, Collaboration of Observational HIV Epidemiological Research Europe (COHERE) study in EuroCoord

**Affiliations:** 1 Department of Infection and Population Health, University College London, London, United Kingdom; 2 Copenhagen HIV programme, University of Copenhagen, Copenhagen, Denmark; 3 Department of Infectious Diseases, Copenhagen University Hospital/Rigshospitalet, Copenhagen, Denmark; 4 Department of Health Sciences, Institute of Infectious Diseases, Milan, Italy; 5 Department of Dermatology, Venerology, and Allergology, St. Josef Hospital, Ruhr-Universität Bochum, Bochum, Germany; 6 CEEISCAT (Agència de Salut Pública de Catalunya) and CIBERESP, Badalona, Catalonia, Spain; 7 Department of Infectious Diseases IRCCS San Raffaele, Milan, Italy; 8 UPMC Université Paris 06, UMR_S 943, Paris, France; 9 INSERM, UMR_S 943, Paris, France; 10 Université of Bordeaux, ISPED, Centre Inserm, U897–Epidémiologie–Biostatistiques, Bordeaux, France; 11 Inserm U897–Epidémiologie–Biostatistiques, Bordeaux, France; 12 Saint-Pierre Hospital, Brussels, Belgium; 13 Universität Köln, Cologne, Germany; 14 Department of Infectious Diseases, Bern University Hospital and University of Bern, Bern, Switzerland; 15 3rd Internal Medicine Department and Infectious Disease Unit, Red Cross General Hospital of Athens, Greece; 16 Université Paris Diderot, Sorbonne Paris Cité, UMR 738, Paris, France; 17 INSERM, UMR 738, Paris, France; 18 Department of Infectious Diseases. University Hospital Ramón y Cajal, IRYCIS, Madrid, Spain; 19 Kings College London School of Medicine, London, United Kingdom; 20 The Kirby Institute, University of New South Wales, Sydney, Australia; 21 Academisch Medisch Centrum bij de Universiteit van Amsterdam, Amsterdam, The Netherlands; 22 Stichting HIV Monitoring, Amsterdam, The Netherlands; 23 German Competence Network for HIV/AIDS, St. Josef Hospital, Ruhr-Universität Bochum, Bochum, Germany; 24 Infectious Diseases Unit, Complejo Hospitalario de Huelva, Spanish VACH Cohort, Spain; 25 University Division of Infectious and Tropical Diseases, University and Spedali Civili of Brescia, Brescia, Italy; 26 Department of Medical and Surgical Sciences, Unit of Infectious Diseases, University “Magna Graecia,” Catanzaro, Italy; 27 INSERM CESP U1018, Université Paris-Sud, AP-HP Public Health Department, Le Kremlin-Bicêtre, France; 28 Medical University Innsbruck, Innsbruck, Austria; 29 Université de Bordeaux, ISPED, Centre INSERM U897-Epidémiologie Statistique, Bordeaux, France; 30 INSERM, ISPED, Centre INSERM U897-Epidémiologie Statistique, Bordeaux, France; Centers for Disease Control and Prevention, United States of America

## Abstract

**Background:**

Few studies have monitored late presentation (LP) of HIV infection over the European continent, including Eastern Europe. Study objectives were to explore the impact of LP on AIDS and mortality.

**Methods and Findings:**

LP was defined in Collaboration of Observational HIV Epidemiological Research Europe (COHERE) as HIV diagnosis with a CD4 count <350/mm^3^ or an AIDS diagnosis within 6 months of HIV diagnosis among persons presenting for care between 1 January 2000 and 30 June 2011. Logistic regression was used to identify factors associated with LP and Poisson regression to explore the impact on AIDS/death. 84,524 individuals from 23 cohorts in 35 countries contributed data; 45,488 were LP (53.8%). LP was highest in heterosexual males (66.1%), Southern European countries (57.0%), and persons originating from Africa (65.1%). LP decreased from 57.3% in 2000 to 51.7% in 2010/2011 (adjusted odds ratio [aOR] 0.96; 95% CI 0.95–0.97). LP decreased over time in both Central and Northern Europe among homosexual men, and male and female heterosexuals, but increased over time for female heterosexuals and male intravenous drug users (IDUs) from Southern Europe and in male and female IDUs from Eastern Europe. 8,187 AIDS/deaths occurred during 327,003 person-years of follow-up. In the first year after HIV diagnosis, LP was associated with over a 13-fold increased incidence of AIDS/death in Southern Europe (adjusted incidence rate ratio [aIRR] 13.02; 95% CI 8.19–20.70) and over a 6-fold increased rate in Eastern Europe (aIRR 6.64; 95% CI 3.55–12.43).

**Conclusions:**

LP has decreased over time across Europe, but remains a significant issue in the region in all HIV exposure groups. LP increased in male IDUs and female heterosexuals from Southern Europe and IDUs in Eastern Europe. LP was associated with an increased rate of AIDS/deaths, particularly in the first year after HIV diagnosis, with significant variation across Europe. Earlier and more widespread testing, timely referrals after testing positive, and improved retention in care strategies are required to further reduce the incidence of LP.

*Please see later in the article for the Editors' Summary*

## Abstract 2

Amanda Mocroft and colleagues investigate risk factors and health outcomes associated with diagnosis at a late stage of infection in individuals across Europe.

*Please see later in the article for the Editors' Summary*

## Editors' Summary

**Background:**

Every year about 2.5 million people become newly infected with HIV, the virus that causes AIDS. HIV can be transmitted through unprotected sex with an infected partner, from an HIV-positive mother to her unborn baby, or through injection of drugs. Most people do not become ill immediately after infection with HIV although some develop a short influenza-like illness. The next stage of the HIV infection, which may last up to 10 years, also has no major symptoms but, during this stage, HIV slowly destroys immune system cells, including CD4 cells, a type of lymphocyte. Eventually, when the immune system is unable to fight off infections by other disease-causing organisms, HIV-positive people develop AIDS-defining conditions—unusual viral, bacterial, and fungal infections and unusual tumors. Progression to AIDS occurs when any severe AIDS-defining condition is diagnosed, when the CD4 count in the blood falls below 200 cells/mm^3^, or when CD4 cells account for fewer than 15% of lymphocytes.

**Why Was This Study Done?:**

People need to know they are HIV positive as soon as possible after they become infected because antiretroviral therapy, which controls but does not cure HIV infection, works best if it is initiated when people still have a relatively high CD4 count. Early diagnosis also reduces the risk of onward HIV transmission. However, 40%–60% of HIV-positive individuals in developed countries are not diagnosed until they have a low CD4 count or an AIDS-defining illness. Reasons for such late presentation include fear of discrimination or stigmatization, limited knowledge about HIV risk factors, testing, and treatment together with missed opportunities to offer an HIV test. Policy makers involved in national and international HIV control programs need detailed information about patterns of late presentation before they can make informed decisions about how to reduce this problem. In this study, therefore, the researchers use data collected by the Collaboration of Observational HIV Epidemiological Research in Europe (COHERE) to analyze trends in late presentation over time across Europe and in different groups of people at risk of HIV infection and to investigate the clinical consequences of late presentation.

**What Did the Researchers Do and Find?:**

The researchers analyzed data collected from 84,524 individuals participating in more than 20 observational studies that were undertaken in 35 European countries and that investigated outcomes among HIV-positive people. Nearly 54% of the participants were late presenters—individuals who had a CD4 count of less than 350 cells/mm^3^ or an AIDS-defining illness within 6 months of HIV diagnosis. Late presentation was highest among heterosexual males, in Southern European countries, and among people originating in Africa. Overall, late presentation decreased from 57.3% in 2000 to 51.7% in 2010/11. However, whereas late presentation decreased over time among men having sex with men in Central and Northern Europe, for example, it increased over time among female heterosexuals in Southern Europe. Finally, among the 8,000 individuals who developed a new AIDS-defining illness or died during follow-up, compared to non-late presentation, late presentation was associated with an increased incidence of AIDS/death in all regions of Europe during the first and second year after HIV diagnosis (but not in later years); the largest increase in incidence (13-fold) occurred during the first year after diagnosis in Southern Europe.

**What Do These Findings Mean?:**

These findings indicate that, although late presentation with HIV infection has decreased in recent years, it remains an important issue across Europe and in all groups of people at risk of HIV infection. They also show that individuals presenting late have a worse clinical outlook, particularly in the first and second year after diagnosis compared to non-late presenters. Several aspects of the study design may affect the accuracy and usefulness of these findings, however. For example, some of the study participants recorded as late presenters may have been people who were aware of their HIV status but who chose not to seek care for HIV infection, or may have been seen in the health care system prior to HIV diagnosis without being offered an HIV test. Delayed entry into care and late presentation are likely to have different risk factors, a possibility that needs to be studied further. Despite this and other study limitations, these findings nevertheless suggest that HIV testing strategies that encourage early testing in all populations at risk, that ensure timely referrals, and that improve retention in care are required to further reduce the incidence of late presentation with HIV infection in Europe.

**Additional Information:**

Please access these websites via the online version of this summary at http://dx.doi.org/ 10.1371/journal.pmed.1001510.

- Information is available from the US National Institute of Allergy and infectious diseases on HIV infection and AIDS
- NAM/aidsmap provides basic information about HIV/AIDS, and summaries of recent research findings on HIV care and treatment
- Information is available from Avert, an international AIDS charity, on many aspects of HIV/AIDS, including detailed information on the stages of HIV infection and on HIV and AIDS in Europe (in English and Spanish)
- The HIV in Europe Initiative has information about strategies to improve earlier diagnosis and access to care in Europe
- Information about COHERE, which was established in 2005 to conduct epidemiological research on the prognosis and outcome of HIV-infected people from across Europe, is available; more information on the consensus definition of late presentation used in this study is available through the HIV in Europe initiative
- Patient stories about living with HIV/AIDS are available through Avert and through the nonprofit website Healthtalkonline

## Introduction

Approximately 40%–60% of HIV-positive persons in developed countries continue to be diagnosed with HIV at a late stage of infection [1]. According to the latest consensus definition from the HIV in Europe study group, late presenters are defined as persons presenting to a clinic that can prescribe antiretroviral therapy (ART) with a CD4 count of less than 350/mm^3^ or an AIDS defining illness [2], the current World Health Organization's recommended threshold for initiation of ART [3]. Late presentation, which may reflect late diagnosis and/or late entry into care, has consequences both for the individual, in terms of poorer outcomes [4]–[6], and for society in terms of the risk of onward transmission due to uncontrolled viremia and lack of awareness of HIV serostatus [7],[8]. Late presentation also has an economic impact, with higher resource use, particularly in the first few months following presentation [9],[10]. Barriers to testing from the patients perspective include concerns about the impact of a positive result, fears around discrimination, confidentiality, criminalisation of risk behaviours, and limited knowledge about accessing testing or treatment on testing positive [11]. Reported concerns by clinicians include language barriers, the belief that lengthy counselling is required, worry about informing individuals of a HIV-positive test result, and lack of knowledge about HIV and potential risk behaviours [12].

Many studies have presented data on late presentation, generally focusing on a specific country or region within a country and applying various definitions for late presentation. Data from Eastern Europe is particularly scarce and hence a Europe-wide perspective of late presentation using uniform definitions can help inform policy and decision making on future continent-wide HIV testing strategies. Such results will provide a platform for future monitoring of late presentation following appropriate interventions.

The Collaboration of Observational HIV Epidemiological Research Europe (COHERE) study provides a unique opportunity to describe the epidemiology of those testing HIV positive at a late stage of HIV infection compared to those testing earlier, and to look at regional differences within HIV exposure groups. The aims of the present analyses were to describe trends in late presentation over time in different regions of Europe and according to HIV exposure group, and to investigate the clinical consequences of late presentation.

## Methods

### Patients

COHERE is a collaboration of 33 cohorts from across Europe and is part of the EuroCoord network (www.EuroCoord.net). COHERE was established in 2005 with the aim of conducting epidemiological research on the prognosis and outcome of HIV-positive persons, which the individual contributing cohorts cannot address themselves because of sample size or heterogeneity of specific subgroups of HIV-positive persons. Local ethical committee and/or other regulatory approval were obtained as applicable according to local and/or national regulations in all participating cohorts unless no such requirement applied to observational studies according to national regulations. Each cohort submits data using the standardized HIV Collaboration Data Exchange Protocol (HICDEP) [13], including information on patient demographics, use of cART, CD4 counts, AIDS, and deaths. Further details can be found at http://www.eurocoord.net/partners/founding_networks/cohere.aspx.

Twenty three cohorts across 35 European countries provided data for the present analysis. All persons aged ≥16 y, who presented for care (earliest of HIV diagnosis, first clinic visit, or enrolment into the participating cohort) for the first time after 1 January 2000 until 1 January 2011 were included. Baseline was defined as the date of first HIV diagnosis, or the earliest of first clinic visit or cohort enrolment if date of HIV diagnosis was missing, referred to as date of HIV diagnosis. Persons were excluded if gender or HIV diagnosis was missing, if aged <16 y, or where there was evidence of an earlier HIV diagnosis (CD4 count, AIDS diagnosis, or starting ART) more than 1 mo prior to first clinic visit, as were persons from Argentinean centres in EuroSIDA and persons from the seroconverter cohorts in COHERE, because by definition, such persons are not presenting late.

Late presentation was defined as a person diagnosed with HIV with a CD4 count below 350/mm^3^ or an AIDS defining event regardless of the CD4 count, in the 6 mo following HIV diagnosis. Late presentation with advanced disease was defined as a person diagnosed with HIV with a CD4 count below 200/mm^3^or an AIDS defining event, regardless of CD4 cell count, in the 6 mo following HIV diagnosis [2]. Delayed entry into care was defined as ≥3 mo between HIV diagnosis and first clinic visit, in those where both dates were recorded. All persons were required to have at least one CD4 count measured in the 6 mo following diagnosis.

### Statistical Methods

Baseline characteristics of late presenters were compared to non-late presenters and logistic regression was used to identify factors associated with late presentation and late presentation with advanced disease. Factors investigated were age, HIV-exposure group (males having sex with males [MSM], heterosexual male, heterosexual female, male intravenous drug user [IDU], female IDU, other [including patients with unknown HIV exposure group, likely to include a number of IDUs, MSMs, and heterosexuals]), region of origin (Europe, Africa, other [including patients from Central/Southern America and Asia], unknown), European region of HIV diagnosis (based on the cohort location and defined as: South: Greece, Israel, Italy, Portugal, and Spain; Central: Austria, Belgium, France, Germany, Luxemburg, and Switzerland; North: Denmark, Finland, Ireland, Netherlands, Norway, Sweden, and UK; East: Belarus, Bosnia and Herzegovina, Bulgaria, Croatia, Czech Republic, Estonia, Hungary, Latvia, Lithuania, Poland, Romania, Russia, Serbia, Slovakia, Slovenia, and Ukraine), calendar year of diagnosis, and delayed entry into care. *A priori*, we were interested in comparing changes over time within European regions and HIV exposure groups, and performed formal tests of interaction before presenting the stratified analyses. Logistic regression was also used to identify characteristics associated with delayed entry into care among late presenters.

Kaplan Meier survival analysis was used to estimate the probability of the composite endpoint of a new AIDS defining event/deaths and Poisson regression models to evaluate the incidence of AIDS/death. Models were adjusted for the same factors as above, and additionally the effect of time after HIV diagnosis (<1, 1–2, >2 y). Recurrent AIDS events were not collected by all participating cohorts and thus were not included.

Different sensitivity analyses were performed to assess the robustness of our findings. Analyses were repeated excluding persons where date of HIV infection was not available in the dataset and excluding persons with known delayed entry into care. We also assessed how the proportion of late presenters changed by using a different time window around the CD4 count at HIV diagnosis (1 mo, 12 mo) and also assuming those without a CD4 count measured were late presenters or not.

All analyses were performed using Statistical Analysis Software Version 9.3 (Statistical Analysis Software).

## Results

Of 107,735 persons in COHERE diagnosed with HIV after 1 January 2000, 84,524 were included in these analyses (78.5%; Table S1). Persons were primarily excluded because they lacked information on CD4 count at HIV diagnosis. Excluded persons were more likely to be from “other” HIV exposure groups (i.e., not categorised as MSM, IDU, or heterosexual), from Eastern Europe, younger, to have no prior AIDS diagnosis at diagnosis, diagnosed in earlier calendar years, and less likely to die. Characteristics of the 84,524 included persons are shown in Table 1; 45,488 persons were classified as late presenters (53.8%) and 28,081 (33.2%) as late presenters with advanced disease. The highest proportion of late presenters was among heterosexual males (66.1%; 11,158/16,875), persons from clinics in Southern Europe (57.0%; 4,447/7,796), and persons originating from Africa (65.1%; 7,708/11,833). 11,903 of the late presenters had AIDS at HIV-1 diagnosis (20.6%), the most common diagnoses were *Pneumocystis jiroveci* pneumonia (20.6%; 2,457/11,903), cytomegalovirus (excluding retinitis, 13.9%; 1,657/11,903), and oesophageal candidiasis (12.3%; 1,463/11,903). Among the 34,561 persons with data on both date of HIV diagnosis and first contact with a clinic, 7.9% (2,720/34,561) had delayed entry into care. Among late presenters, 7.4% had delayed entry into care (1,408/18,933); such individuals were younger (adjusted odds ratio (aOR 0.87/10 y older; 95% CI 0.83–0.91), presented later (aOR 0.96/year later; 95% CI 0.94–0.97), and were more likely to be under care in Southern Europe compared to Northern Europe (aOR 2.02; 95% CI 1.77–2.30). HIV exposure group or region of origin were not associated with delayed entry into care among late presenters.

**Table 1:** Characteristics at HIV diagnosis of late presenters and late presenters with advanced disease: COHERE 2000–2011.

Characteristics	Subcharacteristics	All		Late Presenters		Late Presents with Advanced Disease	
		*n*	Percent	*n*	Percent	*n*	Percent
All		84,524	100	45,488	53.8	28,081	33.2
HIV exposure group	MSMs	32,761	38.8	14,317	43.7	7,924	24.2
	Male heterosexual	16,875	20.0	11,158	66.1	7,723	45.8
	Female heterosexual	20,131	23.8	11,479	57.0	6,788	33.7
	Male IDU	4,142	4.9	2,393	57.8	1,651	39.9
	Female IDU	1,413	1.7	718	50.8	453	32.1
	Male other	6,312	7.5	3,678	58.3	2,435	38.6
	Female other	2,890	3.4	1,745	60.4	1,107	38.3
European region of care	South	7,796	9.2	4,447	57.0	2,873	36.9
	Central	39,949	47.3	21,729	54.4	13,192	33.0
	North	35,583	42.1	18,748	52.7	11,673	32.8
	East	1,196	1.4	564	47.2	343	28.7
Region of origin	Europe	31,370	37.1	16,163	51.5	10,394	33.1
	Africa	11,833	14.0	7,708	65.1	4,770	40.3
	Other	5,812	6.9	3,348	57.6	2,157	37.1
	Unknown	35,509	42.0	18,269	51.5	10,760	30.3
Delayed entry into care^a^		2,720	7.9	1,408	7.4	767	6.6
		Median	IQR	Median	IQR	Median	IQR
Age	Years	36	30–43	38	31–45	39	32–46
CD4 count	/mm^3^	333	160–523	175	70–270	94	36–160
Baseline	Month/year	5/05	10/02–11/07	2/05	8/02–8/07	11/04	6/02–6/07

Late presentation: diagnosed with HIV with a CD4 count below 350/mm^3^ or an AIDS defining event regardless of the CD4 count, in the 6 mo following HIV diagnosis. Late presentation with advanced disease: diagnosed with HIV with a CD4 count below 200/mm^3^or an AIDS defining event, regardless of CD4 cell count, in the 6 mo following HIV diagnosis.

a Delayed entry into care: ≥3 mo between HIV diagnosis and first clinic visit, in those patients where both dates were recorded (*n* = 34,561). Baseline was defined as the earliest of HIV test, first study visit, or cohort enrolment. “Other” regions included Central/Southern America (*n* = 4,277) and Asia (*n* = 1,005). “Other” HIV male and female transmission groups included 5,350 (84.8%) and 2,046 (70.8%) patients with unknown HIV-exposure group, respectively, likely to include a number of IDUs, MSMs, and heterosexuals.

IQR, interquartile range.

**Figure 1:**
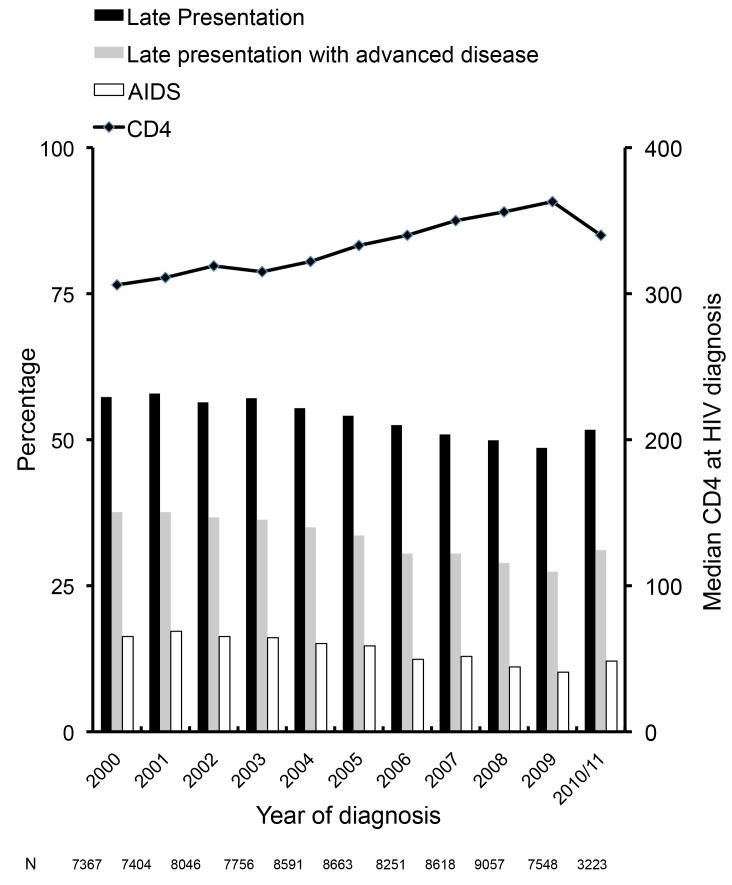
Changes over time in late presentation and CD4 count at HIV diagnosis: COHERE 2000–2011. Late presentation: diagnosed with HIV with a CD4 count below 350/mm^3^ or an AIDS defining event regardless of the CD4 count, in the 6 mo following HIV diagnosis. Late presentation with advanced disease: diagnosed with HIV with a CD4 count below 200/mm^3^or an AIDS defining event, regardless of CD4 cell count, in the 6 mo following HIV diagnosis.

Late presentation declined from 57.3% (4,222/7,367) in 2000 to 51.7% (1,665/3,223) in 2010/2011 (Figure 1). The median CD4 count at diagnosis among all persons increased from 306/mm^3^ in 2000 to 363/mm^3^ in 2009. Factors associated with late presentation included older age (aOR 1.41/10 y older; 95% CI 1.39–1.43), and region of origin, with persons from Africa (aOR 1.75; 95% CI 1.66–1.84) and persons from other regions (aOR 1.40; 95% CI 1.32–1.48) having higher odds of late presentation compared to persons originating from Europe. Compared to persons from Central Europe, persons under care in Southern Europe (aOR 1.41; 95% CI 1.33–1.48) had higher odds of late presentation, as did all HIV-exposure groups compared to MSM. This ranged from over a doubling in heterosexual men (aOR 2.05; 95% CI 1.97–2.14) to 31% for female IDUs (aOR 1.31; 95% CI 1.18–1.46). Delayed entry into care was associated with decreased odds of late presentation (aOR 0.91; 95% CI 0.84–0.99). After adjustment for these confounders, there was a 4% decrease in the odds of late presentation per later calendar year (aOR 0.96; 95% CI 0.96–0.97). Results were similar for late presentation with advanced disease (Figure 1), with a decrease in advanced disease at presentation of 5% per year after adjustment (aOR 0.95; 95% CI 0.94–0.95). Results were highly consistent excluding patients with unknown or delayed entry into care (Table S2).

**Figure 2:**
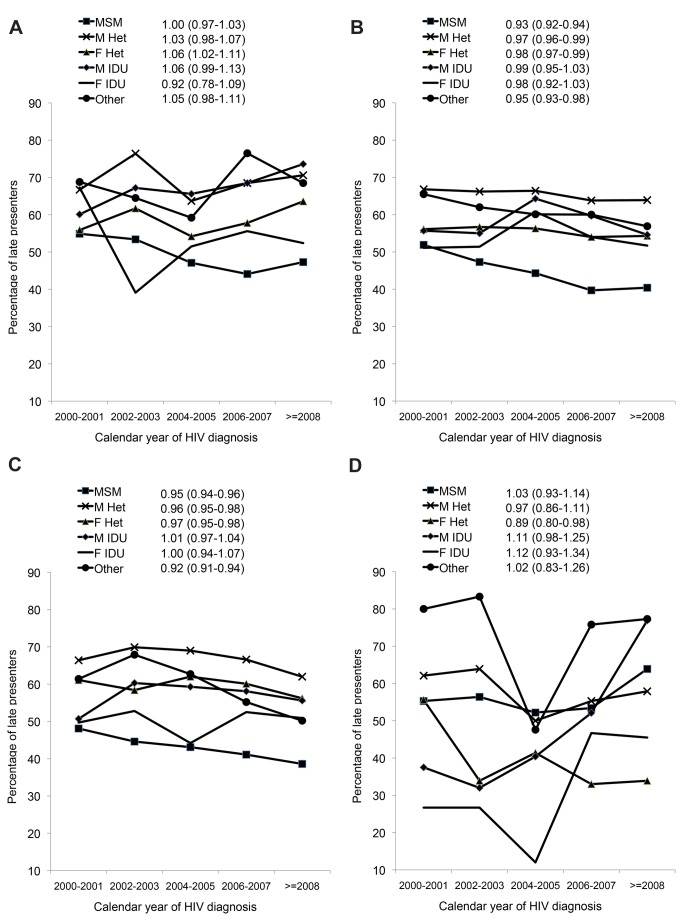
(A–D) Changes in late presentation over calendar time; stratified by HIV exposure group: COHERE 2000–2011. (A) Southern Europe. (B) Central Europe. (C) Northern Europe. (D) Eastern Europe. All models adjusted for age, delayed entry into care (≥3 mo) after HIV diagnosis, region of origin, European region of care, and HIV mode of infection. F, female; Het, heterosexual; M, male.

Figure 2 shows the crude percentage of late presenters within HIV-exposure groups, region, and calendar period, and the aOR of late presentation per year of later HIV diagnosis (also shown in Table S3). Later calendar year was associated with a decrease in late presentation among MSM, and male and female heterosexuals in both Central and Northern Europe (Figure 2B and 2C). For example, the proportion of MSMs with late presentation in Central Europe declined from 51.9% (1,201/2,312) in 2000/2001 to 40.4% (1,352/3,348)≥2008 (aOR 0.93 per year later; 95% CI 0.92–0.94) and from 48.1% (919/1,911) to 38.6% (1,731/4,484) in Northern Europe (Figure 2C; aOR 0.95 per year later; 95% CI 0.94–0.96). Late presentation also decreased over time in heterosexual females from Eastern Europe from 55.6% (25/45) in 2000/2001 to 33.9% (20/59)≥2008 (aOR 0.89 per year later; 95% CI 0.80–0.98). Late presentation increased in male IDUs from Southern Europe (Figure 2A; aOR 1.06 per year later; 95% CI 0.99–1.13), and also among male and female IDUs from Eastern Europe, although the aORs failed to reach statistical significance, possibly owing to the smaller number of patients from Eastern Europe.

Results for presentation with advanced disease are summarised in Table 2. Within Southern Europe, late presentation with advanced disease decreased in MSMs from 36.5% (107/293) during 2000/2001 to 29.7% (364/1,226)≥2008 (aOR 0.94 per year later; 95% CI 0.92–0.97), but was stable in other infection groups. In Central Europe late presentation with advanced disease was decreasing in all HIV-exposure groups except male IDUs, and most rapidly in MSMs, from 33.0% (763/2,312) to 20.7% (693/3,348; aOR 0.91 per year later; 95% CI 0.90–0.92). Similar results were seen in Northern Europe, with a decrease over time in all HIV-exposure groups except male and female IDUs. There were no significant changes over time in Eastern Europe in the odds of late presentation with advanced disease.

**Table 2:** Percentage of late presenters with advanced disease and adjusted odds of late presentation with advanced disease associated with later calendar years of HIV diagnosis: a comparison across regions and HIV exposure group.

Region of Care	HIV Exposure Group	2000–2001		2002–2003		2004–2005		2006–2007		≥2008		Per Year Later HIV Diagnosis			
												Univariate		Multivariate	
		*n*	Percent	*n*	Percent	*n*	Percent	*n*	Percent	*n*	Percent	OR (95% CI)	*p*-Value	aOR^a^ (95% CI)	*p*-Value
South	MSM	293	36.5	369	37.4	635	28.7	919	25.8	1,226	22.9	0.92 (0.89–0.94)	<0.0001	0.94 (0.92–0.97)	<0.0001
	M Het	198	51.0	203	54.7	281	47.7	384	48.4	361	49.6	0.98 (0.95–1.02)	0.41	1.00 (0.96–1.04)	0.82
	F Het	222	38.3	188	36.2	325	35.9	313	34.8	352	41.2	1.02 (0.98–1.06)	0.28	1.03 (0.99–1.07)	0.15
	M IDU	218	42.2	122	48.4	160	48.8	143	46.2	106	50.0	1.03 (0.98–1.09)	0.20	1.00 (0.94–1.07)	0.92
	F IDU	52	40.4	23	13.0	33	30.3	27	33.3	21	38.1	1.01 (0.90–1.13)	0.88	0.95 (0.80–1.13)	0.55
	Other	112	55.4	124	46.8	125	37.6	115	53.9	146	45.9	0.99 (0.94–1.04)	0.73	1.01 (0.95–1.07)	0.83
Central	MSM	2,312	33.0	2,460	27.8	2,912	25.1	3,070	20.1	3,348	20.7	0.92 (0.91–0.93)	<0.0001	0.91 (0.90–0.92)	<0.0001
	M Het	1,949	44.8	2,078	46.4	1,910	43.3	1,570	42.4	1,425	41.7	0.98 (0.96–0.99)	0.0041	0.97 (0.96–0.98)	<0.0001
	F Het	2,359	31.8	2,705	33.0	2,407	32.0	1,915	28.9	1,499	29.6	0.98 (0.97–1.00)	0.024	0.97 (0.95–0.98)	<0.0001
	M IDU	469	36.5	351	36.5	339	44.5	236	37.7	207	39.1	1.01 (0.98–1.05)	0.43	0.99 (0.96–1.03)	0.80
	F IDU	184	35.3	146	30.8	120	42.5	113	32.7	118	28.0	0.98 (0.93–1.04)	0.50	0.94 (0.89–0.99)	0.031
	Other	832	44.8	819	40.9	828	41.3	662	36.6	606	37.5	0.96 (0.94–0.98)	0.0003	0.95 (0.93–0.98)	<0.0001
North	MSM	1,911	29.3	2,244	25.2	3,042	24.6	3,289	20.6	4,484	19.1	0.94 (0.93–0.95)	<0.0001	0.92 (0.90–0.93)	<0.0001
	M Het	1,064	49.3	1,186	51.0	1,269	51.8	1,184	46.2	1,636	42.2	0.96 (0.95–0.98)	<0.0001	0.95 (0.94–0.97)	<0.0001
	F Het	1,276	39.0	1,566	37.9	1,661	37.9	1,419	36.8	1,576	33.7	0.98 (0.96–0.99)	0.0051	0.96 (0.94–0.97)	<0.0001
	M IDU	460	35.4	343	46.1	280	44.6	234	39.3	243	36.2	1.00 (0.97–1.04)	0.88	0.99 (0.95–1.02)	0.45
	F IDU	151	31.1	106	40.6	86	34.9	80	31.3	57	26.3	0.99 (0.92–1.05)	0.65	0.98 (0.92–1.05)	0.53
	Other	495	44.2	542	47.1	541	40.9	901	36.7	2,257	29.0	0.92 (0.90–0.94)	<0.0001	0.92 (0.90–0.94)	<0.0001
East	MSM	47	44.7	39	43.6	67	26.9	58	25.9	36	36.1	0.91 (0.82–1.01)	0.074	0.90 (0.81–1.01)	0.070
	M Het	29	44.8	36	38.9	46	32.6	47	36.2	19	31.6	0.92 (0.81–1.05)	0.21	0.93 (0.81–1.06)	0.27
	F Het	45	31.1	56	21.4	94	24.5	94	20.2	59	20.3	0.94 (0.85–1.04)	0.26	0.92 (0.82–1.02)	0.13
	M IDU	48	31.3	75	17.3	47	17.0	48	33.3	13	38.5	1.07 (0.94–1.27)	0.30	0.97 (0.85–1.12)	0.68
	F IDU	30	6.7	15	13.3	25	4.0	15	20.0	11	27.3	1.27 (0.98–1.63)	0.068	1.24 (0.94–1.64)	0.14
	Other	15	60.0	6	83.3	21	28.6	33	42.4	22	54.6	0.94 (0.80–1.11)	0.45	0.94 (0.77–1.15)	0.55

a Adjusted additionally for age, region of origin, and delayed entry into care (≥3 mo between HIV diagnosis and first clinic visit). Late presentation with advanced disease: diagnosed with HIV with a CD4 count below 200/mm^3^or an AIDS defining event, regardless of CD4 cell count, in the 6 mo following HIV diagnosis.

F, female; Het, heterosexual; M, male.

**Figure 3:**
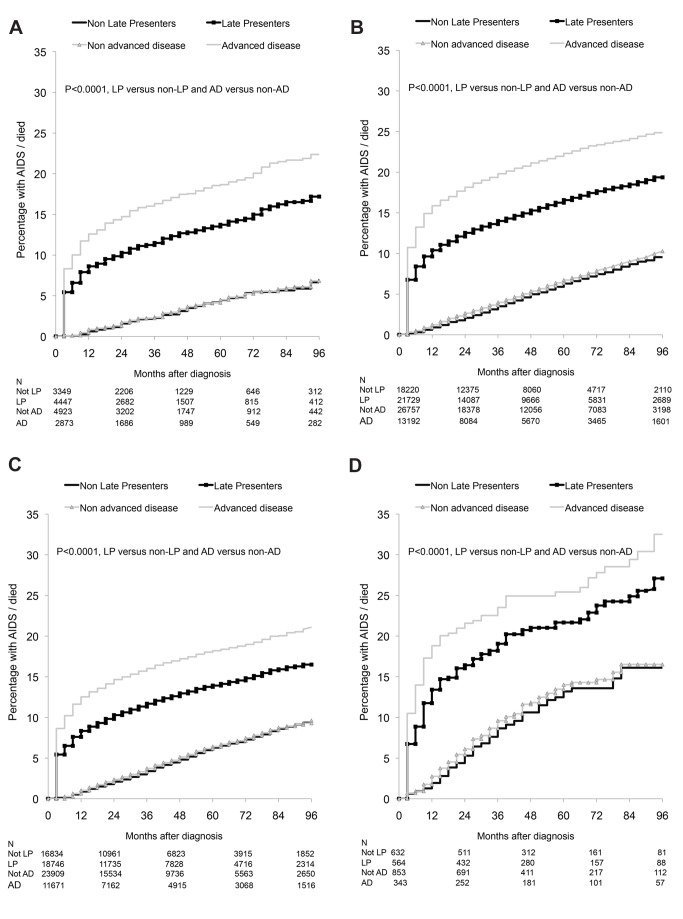
(A–D) Progression to new AIDS/death: role of late presentation or late presentation with advanced disease: COHERE 2000–2011. (A) Southern Europe. (B) Central Europe. (C) Northern Europe. (D) Eastern Europe. LP, diagnosed with HIV with a CD4 count below 350/mm^3^ or an AIDS defining event regardless of the CD4 count, in the 6 mo following HIV diagnosis. Presentation with advanced disease (AD): diagnosed with HIV with a CD4 count below 200/mm^3^ or an AIDS defining event regardless of the CD4 count, in the 6 mo following HIV diagnosis.

There were 8,187 AIDS/deaths (1,852 deaths, 6,283 AIDS, 52 AIDS/death on the same date) during 327,003 person-years of follow-up. The Kaplan-Meier probability of progression to AIDS/death stratified by late presentation and late presentation with advanced disease is shown in Figures 3A–3D. Late presenters or late presenters with advanced disease within each region had a steep increase in probability of new AIDS/death within the first 1 or 2 y following presentation, but followed a similar curve as those who did present late after this period, with some differences between regions (*p*<0.0001, test for interaction).

This finding is explored in Table 3. Late presentation was associated with the highest rate of AIDS/death in the first year after HIV diagnosis among persons from Southern Europe (adjusted incidence rate ratio [aIRR] 13.02; 95% CI 8.19–20.70) and the lowest rate in Eastern Europe (aIRR 6.64; 95% CI 3.55–12.43). In the second year after diagnosis, late presentation was associated with a higher rate of AIDS/death among persons from Southern, Central, and Northern Europe, but was similar in both late and non-late presenters from Eastern Europe. Late presentation was not associated with an increased rate of AIDS/death after the initial 2 y, within any European region of care. Also shown are the results for late presentation with advanced disease, which showed very similar findings. Late presentation was associated with an increased rate of AIDS/death in those presenting with advanced disease beyond 2 y in Southern Europe (aIRR 1.38; 95% CI 1.01–1.88), and only in the first year after diagnosis for those from Eastern Europe (aIRR 6.98; 95% CI 4.22–11.56). Results were similar when excluding patients with unknown or delayed entry into care (Table S4).

**Table 3:** Number and percentage of AIDS/deaths and adjusted incidence rate ratios of AIDS/death after HIV diagnosis in COHERE 2000–2011; late presenters versus non late presenters stratified by European region of care and time since presentation.

*European Region of Care*	*Years since Diagnosis*	*n* (%) AIDS/	Univariate		Multivariate	
		Deaths^a^	IRR (95% CI)	*p*-Value	aIRR (95% CI)	*p*-Value
**Late presenters versus non late presenters**						
South	<1	376 (95.2)	15.83 (9.97–25.10)	<0.0001	13.02 (8.19–20.70)	<0.0001
Central	<1	2,228 (93.5)	12.87 (10.94–15.14)	<0.0001	11.24 (9.54–13.25)	<0.0001
North	<1	1,528 (91.9)	10.70 (8.96–12.75)	<0.0001	9.30 (7.79–11.11)	<0.0001
East	<1	75 (86.2)	7.55 (4.10–13.88)	<0.0001	6.64 (3.55–12.43)	<0.0001
South	1–2	56 (70.0)	1.93 (1.20–3.11)	0.0070	1.55 (0.94–2.53)	0.081
Central	1–2	374 (69.5)	2.03 (1.69–2.43)	<0.0001	1.78 (1.48–2.15)	<0.0001
North	1–2	280 (64.7)	1.72 (1.42–2.10)	<0.0001	1.57 (1.29–1.93)	<0.0001
East	1–2	16 (45.7)	1.02 (0.53–1.99)	0.95	0.94 (0.47–1.91)	0.87
South	>2	112 (64.4)	1.47 (1.08–2.01)	0.015	1.21 (0.88–1.67)	0.24
Central	>2	726 (55.7)	1.04 (0.94–1.16)	0.45	0.95 (0.85–1.06)	0.35
North	>2	535 (52.5)	0.96 (0.84–1.08)	0.47	0.88 (0.78–1.00)	0.049
East	>2	35 (45.5)	0.90 (0.57–1.41)	0.64	0.80 (0.50–1.29)	0.36
**Advanced disease versus non-advanced disease**						
South	<1	356 (90.1)	17.13 (12.31–23.85)	<0.0001	14.63 (10.46–20.47)	<0.0001
Central	<1	2,069 (86.8)	14.96 (13.28–16.84)	<0.0001	13.27 (11.77–14.98)	<0.0001
North	<1	1,437 (86.4)	14.14 (12.29–16.27)	<0.0001	12.45 (10.80–14.37)	<0.0001
East	<1	64 (73.6)	7.85 (4.88–12.65)	<0.0001	6.98 (4.22–11.56)	<0.0001
South	1–2	48 (60.0)	2.89 (1.85–4.52)	<0.0001	2.31 (1.46–3.68)	0.0004
Central	1–2	243 (45.2)	1.88 (1.59–2.23)	<0.0001	1.62 (1.36–1.93)	<0.0001
North	1–2	194 (44.8)	1.80 (1.49–2.17)	<0.0001	1.61 (1.32–1.96)	<0.0001
East	1–2	9 (25.7)	0.99 (0.46–2.11)	0.97	0.87 (0.39–1.94)	0.73
South	>2	86 (49.7)	1.72 (1.28–2.32)	0.0004	1.38 (1.01–1.88)	0.041
Central	>2	463 (35.5)	1.17 (1.05–1.31)	0.0061	1.06 (0.94–1.19)	0.32
North	>2	351 (34.4)	1.02 (0.90–1.17)	0.72	0.94 (0.82–1.07)	0.33
East	>2	21 (27.3)	0.86 (0.52–1.41)	0.54	0.75 (0.44–1.29)	0.30

Adjusted additionally for age, region of origin, delayed entry into care (≥3 mo between HIV diagnosis and first clinic visit), and HIV-exposure group. Late presentation: presenting for care with a CD4 count below 350/mm^3^ or presenting with an AIDS defining event regardless of the CD4 count, in the 6 mo following presentation. Advanced disease: presenting for care with a CD4 count below 200/mm^3^or presenting with an AIDS defining event, regardless of CD4 cell count, in the 6 mo following presentation.

a Figures are *n* (%) of clinical events (AIDS/deaths) in late presenters or late presenters with advanced disease.

The definition of late presentation or presentation with advanced disease required that a CD4 count was measured within 6 mo of diagnosis. Table S5 considers the odds of having missing CD4 count data, which was consistently higher in Eastern Europe compared to Southern Europe in all HIV-exposure groups. The proportion of late presenters and late presenters with advanced disease varied quite widely, especially in Eastern Europe, as shown in sensitivity analyses in Table 4. Changing the requirement for the CD4 count to be measured within 1 mo or 12 mo of diagnosis did not greatly alter the proportion of late presenters or late presenters with advanced disease, while assuming those without a CD4 count were late presenters increased the proportion of late presenters to 61.9% overall (63,467/102,532) and 73.8% in Eastern Europe (1,781/2,413). Conversely, assuming that those without a CD4 count and without AIDS were not late presenters lowered the proportion of late presenters to 45.1% overall (46,242/102,532) and to 26.0% in Eastern Europe (627/2,413).

**Table 4:** Sensitivity analyses showing the proportion of late presenters or late presenters with advanced disease using different inclusion criteria for CD4 count at HIV diagnosis: COHERE 2000–2011.

Characteristic	Region	*n* Late Presenters and Presenters with Advanced Disease	CD4 Measured within *x* Months of HIV Diagnosis			First CD4, Irrespective of Date	No CD4 in First 6 mo = LP/AD	No CD4 in First 6 mo Not LP/AD
			*x* = 1	*x* = 6^a^	*x* = 12			
All patients		Total *n*	67,338	84,524	88,507	98,743	102,532	102,532
		LP (%)	54.7	53.8	53.5	52.6	61.9	45.1
		AD (%)	34.5	33.2	32.7	31.4	45.0	28.1
European region of care	South	Total *n*	5,491	7796	8,122	8,788	8,937	8,937
		LP (%)	58.2	57.0	56.8	56.9	62.5	50.5
		AD (%)	38.9	36.9	36.4	36.0	44.9	32.9
	Central	Total *n*	28,491	39,949	42,587	50,096	50,729	50,729
		LP (%)	56.1	54.4	53.9	52.5	64.1	43.5
		AD (%)	35.3	33.0	32.2	30.3	47.3	26.7
	North	Total *n*	17,300	35,583	36,330	37,488	40,453	40,453
		LP (%)	53.0	52.7	52.5	52.2	68.4	47.0
		AD (%)	33.0	32.8	32.6	32.2	40.9	29.5
	East	Total *n*	724	1,196	1,468	2,371	2413	2,413
		LP (%)	48.2	47.2	46.1	45.3	73.8	26.0
		AD (%)	30.8	28.7	27.3	23.7	64.7	16.9

Late presentation: diagnosed with HIV with a CD4 count below 350/mm^3^ or an AIDS defining event regardless of the CD4 count, in the 6 mo following HIV diagnosis. Late presentation with advanced disease: diagnosed with HIV with a CD4 count below 200/mm^3^or an AIDS defining event, regardless of CD4 cell count, in the 6 mo following HIV diagnosis.

a Corresponds to main analyses presented in Results.

AD, advanced disease; LP, late presenter.

## Discussion

This study included almost 85,000 persons diagnosed with HIV across Europe after 1 January 2000 and found over half were late presenters and one-third presented with advanced disease using the HIV in Europe definition [2]. The study focused on regional differences within HIV exposure groups, providing important information for health care providers and for future testing initiatives. Overall, late presentation decreased, and especially in MSMs. However, late presentation remains a significant issue across Europe and in all HIV exposure groups. Late presentation increased in male IDUs and female heterosexuals from Southern and IDUs in Eastern Europe. Late presentation was associated with an increased rate of AIDS/deaths, particularly in the first year after HIV diagnosis, although this also varied across Europe. Earlier and more widespread testing, timely referrals after testing positive, as well as improved retention in care strategies are required to further reduce the incidence of late presentation.

The prevalence of late presentation was similar to that reported from other recent European studies [14]–[17], as is the decrease in late presentation [18],[19]. Our study is unique in its ability to report continent-wide changes within HIV exposure groups over a significant period of time. The decreases in late presentation are likely associated with changes to provider initiated HIV testing policies and the massive scale-up of antenatal screening for HIV [20],[21]. Factors associated with late presentation in this and previous studies included non-MSMs HIV-exposure group [1],[5],[14],[16],[17],[22], older age [1],[14],[16]–[19],[23], and non-European origin [14],[17],[19],[22],[24]. Reasons for the increase in late presentation among IDUs in Eastern Europe and heterosexual females and male IDUs from Southern Europe may include suboptimal health care offered to these populations, differences in characteristics that we were unable to adjust for, such as socioeconomic status, the pattern of the underlying epidemic, and appearance of symptoms that may ultimately promote presentation for care [25],[26]. Not all persons recommended for testing undergo testing for a variety of reasons, including stigmatisation, discrimination, criminalisation laws, lack of knowledge, or perceived risk [12]. Not testing inevitably leads to later presentation and initiation of ART, despite the fact that an alarmingly high proportion of the late presenters have been in contact with the health system in the years preceding HIV diagnosis [19],[27].

As reported by others, late presentation or presentation with advanced disease was associated with a significantly increased incidence of AIDS/deaths [28],[29]. We found an increased incidence of AIDS/death, particularly in the first year after HIV diagnosis [6],[18],[30]. There was also significant regional variation, with a 13-fold increased rate in persons in care in Southern Europe and a 6-fold increased rate in Eastern Europe. There was a small increased rate of AIDS/death associated with late presentation in the second year after diagnosis, with the exception of Eastern Europe, and no differences after this time. Morbidity and mortality among HIV-positive individuals in Eastern Europe has been reported to be particularly poor in EuroSIDA [31], the only contributor to COHERE with data from Eastern Europe. Unfortunately, COHERE does not have additional data on factors such as whether individuals are current IDUs, on opiate substitution therapy, or alcohol dependant, all of which may contribute to clinical progression after HIV diagnosis and which likely vary significantly across regions.

Collaborations such as the HIV in Europe initiative have helped raise awareness of HIV testing, and developed an indicator disease testing strategy, whereby all persons presenting for care for a number of indicator diseases are routinely tested for HIV [27]. Despite this and other strategies, individuals continue to test late for HIV with consequences in terms of a poor prognosis, an increased risk of onward HIV transmission, increased costs to the health system, and suboptimal benefits of ART [2],[32]. Increasing awareness of HIV risk factors, increased opportunities for testing, reducing barriers to testing, and increasing the availability of effective ART will help address ongoing HIV transmission across Europe, and especially in Eastern Europe where the number of newly diagnosed infections continues to be very high [33].

Persons were required to have a CD4 count measured within 6 mo of presentation to be included in analyses. If those with missing CD4 counts were assumed late presenters, the proportion of late presenters rose to 62% overall and 74% in Eastern Europe, with 45% overall and 65% from Eastern Europe having advanced disease. Accurate information on CD4 count at presentation is essential for monitoring late presentation, a recent report suggested 18 of 28 countries routinely monitored CD4 count at presentation, and only 11 had information on at least 50% of persons testing HIV positive [34]. Missing CD4 counts were consistently more likely in persons from Eastern Europe (Table S4) among all HIV exposure categories. Persons from Eastern Europe were exclusively included from the EuroSIDA cohort (details at www.cphiv.dk). Participating sites are typically centres of excellence, likely not representative of all persons in the region [35]. Further, inclusion criteria include a pre-booked clinic outpatient appointment, meaning persons have to survive long enough to be recruited to EuroSIDA, which may have excluded a number of late presenters. Persons from Eastern Europe are known to have a poor prognosis [31], and CD4 count may be more likely to be missing in those who die before a CD4 count is measured [36]. As a result, we are likely underestimating the proportion of and consequences of late presentation in this region.

Some limitations of the study should be noted. Date of HIV infection was assumed to be the date of first clinic visit or enrolment into the participating cohort. We performed sensitivity analyses excluding patients where HIV-test date was not reported to COHERE with highly consistent results (Tables S2 and S3). Late presentation and delayed entry into care can be thought of as two distinct issues with different risk factors, interventions, and outcomes [1],[19],[37]. Late presentation reflects patients who are unaware of their HIV infection and do not test until their CD4 count has declined, while delayed entry into care reflects persons who are aware of their HIV status but chose not to seek care for their HIV. Less than 10% of our patient population had delayed entry into care, although this information was only available for a minority of patients. We excluded cohorts including only seroconverters from our analyses, because persons in these cohorts are unlikely to be late presenters. However, inclusion of these cohorts did not substantially alter our findings (Table S2). Local regulations meant information on ethnic origin was not available for over 40% of persons, and likely includes a significant number of migrants who are known to present later for testing [32].

In summary, while late presentation has decreased over time across Europe, it remains a significant issue across the European continent with implications for both individuals and the public health in most European regions. Late presentation was associated with an increased rate of AIDS/death, particularly in the first year after HIV diagnosis. It is important that earlier HIV testing strategies are targeted to all populations at risk both within the health care system and in community based programs, to ensure timely referrals after testing positive, improved retention in care strategies, and optimal clinical management and initiation of ART in those testing HIV positive.

## Supporting Information

Table S1 **Summary of patients included and excluded from the COHERE analysis of late presentation across Europe: COHERE 2000–2011.** *Persons were also excluded if gender or HIV diagnosis was missing, if aged <16, or if there was evidence of an earlier HIV diagnosis (CD4 count, AIDS diagnosis, or starting ART) more than 1 mo prior to the date of first clinic visit. ^+^Additionally excluded patients from Argentinean centres.
(DOCX)

Table S2 **Adjusted odds of late presentation among persons with known first clinic visit and without delayed entry into care and including seroconverter cohorts contributing to COHERE: COHERE 2000–2011.**
(DOCX)

Table S3 Percentage of late presenters and adjusted odds* of late presentation associated with later calendar years of HIV diagnosis: a comparison across regions and HIV exposure group. *Adjusted additionally for age, region of origin, and delayed entry into care (≥3 mo between HIV diagnosis and first clinic visit). Late presentation: diagnosed with HIV with a CD4 count below 350/mm^3^or an AIDS defining event, regardless of CD4 cell count, in the 6 mo following HIV diagnosis. F, female; Het, heterosexual; M, male.
(DOCX)

Table S4 Number and percentage of AIDS/deaths and adjusted* incidence rate ratios of AIDS/death after HIV diagnosis in COHERE 2000–2011: late presenters versus non late presenters and late presentation with advanced disease versus non-advanced disease stratified by European region of care and time since presentation; excluding persons with delayed entry into care or unknown first visit date ( ***n*** **= 31,733).** Late presentation: presenting for care with a CD4 count below 350/mm^3^ or presenting with an AIDS defining event regardless of the CD4 count, in the 6 mo following presentation. Advanced disease: presenting for care with a CD4 count below 200/mm^3^or presenting with an AIDS defining event, regardless of CD4 cell count, in the 6 mo following presentation. ∧Figures are *n* (%) of clinical events (AIDS/deaths) in late presenters or late presenters with advanced disease. *Adjusted additionally for age, region of origin, and delayed entry into care (≥3 mo between HIV diagnosis and first clinic visit). + Persons from Northern Europe were also excluded as the majority of persons from Northern Europe had delayed entry into care or unknown first visit date.
(DOCX)

Table S5 **Odds of missing CD4 count in the 6 mo following HIV diagnosis stratified by HIV exposure group: COHERE 2000–2011.** Male and females belonging to the “Other” HIV exposure group were combined into a single category.
(DOCX)

## Abbreviations

aIRR: adjusted incidence rate ratio
aOR: adjusted odds ratio
ART: antiretroviral therapy
COHERE: Collaboration of Observational HIV Epidemiological Research Europe
IDU: intravenous drug user
LP: late presentation
MSM: males having sex with males
