# Supplementary material for: Risk Factors and Outcomes for Late Presentation for HIV-Positive Persons in Europe: Results from the Collaboration of Observational HIV Epidemiological Research Europe Study (COHERE)
Source: PLoS Med. 2013 Sep 3;10(9):e1001510. doi: 10.1371/journal.pmed.1001510 (PMC3796947; doi:10.1371/journal.pmed.1001510)
Supplement: Table S1 — Summary of patients included and excluded from the COHERE analysis of late presentation across Europe: COHERE 2000–2011. *Persons were also excluded if gender or HIV diagnosis was missing, if aged <16, or if there was evidence of an earlier HIV diagnosis (CD4 count, AIDS diagnosis, or starting ART) more than 1 mo prior to the date of first clinic visit. +Additionally excluded patients from Argentinean centres. (DOCX) [file pmed.1001510.s001.docx]

| Cohort | Total N | Excluded : HIV test < 1/1/2000 or other reason* | | Excluded : Seroconverter cohort or missing CD4 | | Included | |
| --- | --- | --- | --- | --- | --- | --- | --- |
|  |  | N | % | N | % | N | % |
| **All** | **265346** | **158011** | **59.6** | **22811** | **8.6** | **84524** | **31.9** |
| AHIVCOS | 1458 | 618 | 42.4 | 116 | 8.0 | 724 | 49.7 |
| AMACS | 3329 | 1519 | 45.6 | 254 | 7.6 | 1556 | 46.7 |
| AQUITAINE | 7964 | 6447 | 81.0 | 384 | 4.8 | 1133 | 14.2 |
| ATHENA | 18323 | 9602 | 52.4 | 474 | 2.6 | 8247 | 45.0 |
| BONN | 1732 | 1077 | 62.2 | 29 | 1.7 | 626 | 36.1 |
| CASCADE | 13058 | 8899 | 68.2 | 4159 | 31.9 | 0 | 0 |
| UK CHIC | 37951 | 19360 | 51.0 | 3373 | 8.9 | 15218 | 40.1 |
| CHIPS | 1669 | 1664 | 99.7 | 0 | 0 | 5 | 0.3 |
| COLOGNE | 3564 | 2238 | 62.8 | 122 | 3.4 | 1204 | 33.8 |
| Co-RIS | 4833 | 1647 | 34.1 | 142 | 2.9 | 3044 | 63.0 |
| DHK | 4753 | 2514 | 52.9 | 158 | 3.3 | 2081 | 43.8 |
| EuroSIDA^+^ | 13521 | 9777 | 72.3 | 1973 | 14.6 | 1771 | 13.1 |
| FHDH | 80112 | 47836 | 59.7 | 6727 | 8.4 | 25549 | 31.9 |
| GEMES | 616 | 0 | 0 | 616 | 100 | 0 | 0 |
| HSR | 2449 | 1454 | 59.4 | 104 | 4.3 | 891 | 36.4 |
| ICONA | 8172 | 4421 | 54.1 | 32 | 0.4 | 3719 | 45.5 |
| KOMPNET | 5726 | 3063 | 53.5 | 1074 | 18.8 | 1589 | 27.8 |
| MASTER | 14037 | 8685 | 61.9 | 79 | 0.6 | 5273 | 37.6 |
| MODENA | 1140 | 523 | 45.9 | 617 | 54.1 | 0 | 0 |
| PISCIS | 11220 | 4830 | 43.1 | 1766 | 15.7 | 4624 | 41.2 |
| SHCS | 16909 | 14139 | 83.6 | 1 | 0.0 | 2769 | 16.4 |
| St. PIERRE | 5501 | 3361 | 61.1 | 588 | 10.7 | 1552 | 28.2 |
| VACH | 7309 | 3721 | 50.9 | 639 | 8.7 | 2949 | 40.4 |
