# Supplementary material for: Risk Factors and Outcomes for Late Presentation for HIV-Positive Persons in Europe: Results from the Collaboration of Observational HIV Epidemiological Research Europe Study (COHERE)
Source: PLoS Med. 2013 Sep 3;10(9):e1001510. doi: 10.1371/journal.pmed.1001510 (PMC3796947; doi:10.1371/journal.pmed.1001510)
Supplement: Table S2 — Adjusted odds of late presentation among persons with known first clinic visit and without delayed entry into care and including seroconverter cohorts contributing to COHERE: COHERE 2000–2011. (DOCX) [file pmed.1001510.s002.docx]

|  |  | Excluding persons with delayed entry into care and unknown first visit date (N=31840) | | As main analysis, including seroconverter cohorts (N=88084) | |
| --- | --- | --- | --- | --- | --- |
|  |  | aOR (95% CI) | P | aOR (95% CI) | P |
| HIV | Males having sex with males | 1.00 | - | 1.00 | - |
| Exposure | Male heterosexual | 2.01 (1.88-2.14) | <0.0001 | 2.07 (1.99-2.15) | <0.0001 |
|  | Female heterosexual | 1.50 (1.41-1.60) | <0.0001 | 1.62 (1.56-1.68) | <0.0001 |
|  | Male injecting drug user | 1.81 (1.58-2.08) | <0.0001 | 1.74 (1.63-1.86) | <0.0001 |
|  | Female injecting drug user | 1.13 (0.87-1.47) | 0.37 | 1.47 (1.32-1.63) | <0.0001 |
|  | Male other | 2.01 (1.81-2.23) | <0.0001 | 1.66 (1.58-1.76) | <0.0001 |
|  | Female other | 1.78 (1.56-2.04) | <0.0001 | 1.92 (1.77-2.08) | <0.0001 |
| European | Central | 1.00 | - | 1.00 | - |
| Region of | South | 1.88 (1.72-2.06) | <0.0001 | 1.54 (1046-1.63) | <0.0001 |
| Care | North | 1.22 (0.82-1.81) | 0.34 | 1.14 (1.10-1.18) | <0.0001 |
|  | East | 1.15 (0.99-1.33) | 0.075 | 1.16 (1.03-1.31) | 0.016 |
| Region of | Europe | 1.00 | - | 1.00 | - |
| Origin | Africa | 1.84 (1.68-2.03) | <0.0001 | 1.85 (1.76-1.95) | <0.0001 |
|  | Other | 1.52 (1.35-1.71) | <0.0001 | 1.50 (1.41-1.59) | <0.0001 |
|  | Unknown | 1.14 (1.05-1.23) | 0.0017 | 1.14 (1.10-1.18) | <0.0001 |
| Age | Per 10 years older | 1.39 (1.36-1.43) | <0.0001 | 1.42 (1.40-1.44) | <0.0001 |
| Year presented | Per year later | 0.97 (0.96-0.98) | <0.0001 | 0.97 (0.96-0.97) | <0.0001 |
| Delayed entry into care | Yes versus No |  |  | 0.91 (0.84-0.99) | 0.020 |
