# Supplementary material for: Risk Factors and Outcomes for Late Presentation for HIV-Positive Persons in Europe: Results from the Collaboration of Observational HIV Epidemiological Research Europe Study (COHERE)
Source: PLoS Med. 2013 Sep 3;10(9):e1001510. doi: 10.1371/journal.pmed.1001510 (PMC3796947; doi:10.1371/journal.pmed.1001510)
Supplement: Table S3 — Percentage of late presenters and adjusted odds* of late presentation associated with later calendar years of HIV diagnosis: a comparison across regions and HIV exposure group. *Adjusted additionally for age, region of origin, and delayed entry into care (≥3 mo between HIV diagnosis and first clinic visit). Late presentation: diagnosed with HIV with a CD4 count below 350/mm3or an AIDS defining event, regardless of CD4 cell count, in the 6 mo following HIV diagnosis. F, female; Het, heterosexual; M, male. (DOCX) [file pmed.1001510.s003.docx]

| Region | HIV exp. group | 2000-2001 | | | 2002-2003 | | | 2004-2005 | | | 2006-2007 | | | >2008 | | | Per year later HIV-diagnosis | | | | | | |  |
| --- | --- | --- | --- | --- | --- | --- | --- | --- | --- | --- | --- | --- | --- | --- | --- | --- | --- | --- | --- | --- | --- | --- | --- | --- |
| Of |  |  |  | |  |  | |  |  | |  |  | |  |  | | Univariate | | | Multivariate | | | |  |
| Care |  | N | % | | N | % | | N | % | | N | % | | N | % | | OR (95% CI) | | p | aOR* (95% CI) | | p | |  |
| South | MSM | 293 | | 55.0 | 369 | | 53.4 | 635 | | 47.1 | 919 | | 44.1 | 1226 | | 47.3 | 0.97 (0.94-0.99) | 0.0099 | | | 1.00 (0.97-1.03) | | 0.97 | |
|  | M Het | 198 | | 66.7 | 203 | | 76.4 | 281 | | 63.7 | 384 | | 68.5 | 361 | | 70.6 | 1.01 (0.97-1.05) | 0.75 | | | 1.03 (0.98-1.07) | | 0.22 | |
|  | F Het | 222 | | 55.9 | 188 | | 61.7 | 325 | | 54.2 | 313 | | 57.8 | 352 | | 63.6 | 1.04 (1.00-1.08) | 0.036 | | | 1.06 (1.02-1.11) | | 0.0059 | |
|  | M IDU | 218 | | 60.1 | 122 | | 67.2 | 160 | | 65.6 | 143 | | 68.5 | 106 | | 73.6 | 1.07 (1.02-1.13) | 0.0069 | | | 1.06 (0.99-1.13) | | 0.11 | |
|  | F IDU | 52 | | 67.3 | 23 | | 39.1 | 33 | | 51.5 | 27 | | 55.6 | 21 | | 52.4 | 0.96 (0.86-1.07) | 0.49 | | | 0.92 (0.78-1.09) | | 0.35 | |
|  | Other | 112 | | 68.6 | 124 | | 64.5 | 125 | | 59.2 | 115 | | 76.5 | 146 | | 68.5 | 1.03 (0.98-1.09) | 0.26 | | | 1.05 (0.98-1.11) | | 0.15 | |
| Central | MSM | 2312 | | 52.0 | 2460 | | 47.3 | 2912 | | 44.3 | 3070 | | 39.7 | 3348 | | 40.4 | 0.94 (0.93-0.96) | <0.0001 | | | 0.93 (0.92-0.94) | | <0.0001 | |
|  | M Het | 1949 | | 66.8 | 2078 | | 66.2 | 1910 | | 66.4 | 1570 | | 63.8 | 1425 | | 63.9 | 0.98 (0.97-1.00) | 0.011 | | | 0.97 (0.96-0.99) | | 0.0012 | |
|  | F Het | 2359 | | 56.1 | 2705 | | 56.8 | 2407 | | 56.3 | 1915 | | 54.1 | 1499 | | 54.3 | 0.99 (0.98-1.00) | 0.13 | | | 0.98 (0.97-0.99) | | 0.0032 | |
|  | M IDU | 469 | | 55.7 | 351 | | 55.0 | 339 | | 64.3 | 236 | | 59.8 | 207 | | 54.6 | 1.01 (0.97-1.04) | 0.65 | | | 0.99 (0.95-1.03) | | 0.53 | |
|  | F IDU | 184 | | 51.1 | 146 | | 51.4 | 120 | | 60.8 | 113 | | 54.0 | 118 | | 51.7 | 1.01 (0.97-1.07) | 0.58 | | | 0.98 (0.92-1.03) | | 0.36 | |
|  | Other | 832 | | 65.5 | 819 | | 62.0 | 828 | | 60.1 | 662 | | 60.0 | 606 | | 56.9 | 0.96 (0.94-0.98) | 0.0003 | | | 0.95 (0.93-0.98) | | <0.0001 | |
| North | MSM | 1911 | | 48.1 | 2244 | | 44.6 | 3042 | | 43.1 | 3289 | | 41.1 | 4484 | | 38.6 | 0.96 (0.95-0.97) | <0.0001 | | | 0.95 (0.94-0.96) | | <0.0001 | |
|  | M Het | 1064 | | 66.5 | 1186 | | 69.9 | 1269 | | 69.0 | 1184 | | 66.6 | 1636 | | 62.0 | 0.97 (0.96-0.99) | 0.0013 | | | 0.96 (0.95-0.98) | | <0.0001 | |
|  | F Het | 1276 | | 61.1 | 1566 | | 58.4 | 1661 | | 62.0 | 1419 | | 60.1 | 1576 | | 56.2 | 0.99 (0.97-1.00) | 0.064 | | | 0.97 (0.95-0.98) | | 0.0002 | |
|  | M IDU | 460 | | 50.7 | 343 | | 60.4 | 280 | | 59.3 | 234 | | 58.1 | 243 | | 55.6 | 1.03 (0.99-1.06) | 0.13 | | | 1.01 (0.97-1.04) | | 0.78 | |
|  | F IDU | 151 | | 49.7 | 106 | | 52.8 | 86 | | 44.2 | 80 | | 52.5 | 57 | | 50.9 | 1.01 (0.96-1.08) | 0.64 | | | 1.00 (0.94-1.07) | | 0.96 | |
|  | Other | 495 | | 61.4 | 542 | | 67.9 | 541 | | 62.7 | 901 | | 55.2 | 2257 | | 50.2 | 0.92 (0.91-0.94) | <0.0001 | | | 0.92 (0.91-0.94) | | <0.0001 | |
| East | MSM | 47 | | 55.3 | 39 | | 56.4 | 67 | | 52.2 | 58 | | 53.5 | 36 | | 63.9 | 1.03 (0.93-1.14) | 0.59 | | | 1.03 (0.93-1.14) | | 0.62 | |
|  | M Het | 29 | | 62.1 | 36 | | 63.9 | 46 | | 50.0 | 47 | | 55.3 | 19 | | 57.9 | 0.95 (0.84-1.08) | 0.44 | | | 0.97 (0.86-1.11) | | 0.66 | |
|  | F Het | 45 | | 55.6 | 56 | | 33.9 | 94 | | 41.5 | 94 | | 33.0 | 59 | | 33.9 | 0.92 (0.84-1.00) | 0.057 | | | 0.89 (0.80-0.98) | | 0.015 | |
|  | M IDU | 48 | | 37.5 | 75 | | 32.0 | 47 | | 40.4 | 48 | | 52.1 | 13 | | 76.9 | 1.18 (1.05-1.32) | 0.0041 | | | 1.11 (0.98-1.25) | | 0.11 | |
|  | F IDU | 30 | | 26.7 | 15 | | 26.7 | 25 | | 12.0 | 15 | | 46.7 | 11 | | 45.5 | 1.14 (0.96-1.36) | 0.14 | | | 1.12 (0.93-1.34) | | 0.24 | |
|  | Other | 15 | | 80.0 | 6 | | 83.3 | 21 | | 47.6 | 33 | | 75.8 | 22 | | 77.3 | 0.97 (0.80-1.17) | 0.72 | | | 1.02 (0.83-1.26) | | 0.87 | |
