# Supplementary material for: Risk Factors and Outcomes for Late Presentation for HIV-Positive Persons in Europe: Results from the Collaboration of Observational HIV Epidemiological Research Europe Study (COHERE)
Source: PLoS Med. 2013 Sep 3;10(9):e1001510. doi: 10.1371/journal.pmed.1001510 (PMC3796947; doi:10.1371/journal.pmed.1001510)
Supplement: Table S4 — Number and percentage of AIDS/deaths and adjusted* incidence rate ratios of AIDS/death after HIV diagnosis in COHERE 2000–2011: late presenters versus non late presenters and late presentation with advanced disease versus non-advanced disease stratified by European region of care and time since presentation; excluding persons with delayed entry into care or unknown first visit date ( n = 31,733). Late presentation: presenting for care with a CD4 count below 350/mm3 or presenting with an AIDS defining event regardless of the CD4 count, in the 6 mo following presentation. Advanced disease: presenting for care with a CD4 count below 200/mm3or presenting with an AIDS defining event, regardless of CD4 cell count, in the 6 mo following presentation. ∧Figures are n (%) of clinical events (AIDS/deaths) in late presenters or late presenters with advanced disease. *Adjusted additionally for age, region of origin, and delayed entry into care (≥3 mo between HIV diagnosis and first clinic visit). + Persons from Northern Europe were also excluded as the majority of persons from Northern Europe had delayed entry into care or unknown first visit date. (DOCX) [file pmed.1001510.s004.docx]

|  |  | N (%) AIDS/ | Univariate | | Multivariate | |
| --- | --- | --- | --- | --- | --- | --- |
|  |  | deaths^ | IRR (95% CI) | P | aIRR (95% CI) | P |
| **Late presenters versus non late presenters** | | | |  |  |  |
| *European region of care* | *Years since diagnosis* |  |  |  |  |  |
| South | <1 | 234 (95.1) | 13.35 (7.48-23.85) | <0.0001 | 11.32 (6.31-20.31) | <0.0001 |
| Central | <1 | 1649 (94.2) | 14.82 (12.13-18.12) | <0.0001 | 13.01 (10.62-15.92) | <0.0001 |
| North^+^ | <1 |  |  |  |  |  |
| East | <1 | 65 (85.5) | 7.46 (3.94-14.14) | <0.0001 | 6.28 (3.26-12.10) | <0.0001 |
| South | 1-2 | 31 (67.4) | 11.50 (0.81-2.79) | 0.19 | 1.31 (0.69-2.46) | 0.41 |
| Central | 1-2 | 246 (69.5) | 2.08 (1.65-2.60) | <0.0001 | 1.79 (1.42-2.56) | <0.0001 |
| North^+^ | 1-2 |  |  |  |  |  |
| East | 1-2 | 12 (41.4) | 0.89 (0.43-1.87) | 0.76 | 0.79 (0.37-1.73) | 0.56 |
| South | >2 | 78 (64.5) | 1.35 (0.93-1.96) | 0.11 | 1.03 (0.70-1.51) | 0.90 |
| Central | >2 | 463 (54.7) | 1.04 (0.90-1.19) | 0.62 | 0.93 (0.81-1.07) | 0.29 |
| North^+^ | >2 |  |  |  |  |  |
| East | >2 | 35 (49.3) | 1.07 (0.67-1.70) | 0.78 | 0.98 (0.60-1.59) | 0.92 |
| **Advanced disease versus non-advanced disease** | | | | |  |  |
| *European region of care* | *Years since diagnosis* |  |  |  |  |  |
| South | <1 | 227 (92.3) | 19.39 (12.14-30.96) | <0.0001 | 17.12 (10.66-27.49) | <0.0001 |
| Central | <1 | 1545 (88.3) | 17.28 (14.93-19.99) | <0.0001 | 15.38 (13.27-17.84) | <0.0001 |
| North^+^ | <1 |  |  |  |  |  |
| East | <1 | 54 (71.1) | 7.49 (4.56-12.30) | <0.0001 | 6.24 (3.6*-10.54) | <0.0001 |
| South | 1-2 | 25 (54.4) | 2.06 (1.15-3.68) | 0.015 | 1.80 (0.98-3.28) | 0.059 |
| Central | 1-2 | 164 (46.3) | 2.02 (1.64-2.49) | <0.0001 | 1.69 (1.36-2.10) | <0.0001 |
| North^+^ | 1-2 |  |  |  |  |  |
| East | 1-2 | 6 (20.7) | 0.80 (0.33-1.96) | 0.62 | 0.67 (0.26-1.73) | 0.41 |
| South | >2 | 58 (47.9) | 1.49 (1.05-2.13) | 0.027 | 1.10 (0.76-1.59) | 0.62 |
| Central | >2 | 300 (35.5) | 1.21 (1.05-1.39) | 0.0086 | 1.07 (0.93-1.24) | 0.34 |
| North^+^ | >2 |  |  |  |  |  |
| East | >2 | 21 (29.6) | 1.00 (0.60-1.67) | 0.99 | 0.90 (0.52-1.55) | 0.70 |
