# Supplementary material for: Risk Factors and Outcomes for Late Presentation for HIV-Positive Persons in Europe: Results from the Collaboration of Observational HIV Epidemiological Research Europe Study (COHERE)
Source: PLoS Med. 2013 Sep 3;10(9):e1001510. doi: 10.1371/journal.pmed.1001510 (PMC3796947; doi:10.1371/journal.pmed.1001510)
Supplement: Table S5 — Odds of missing CD4 count in the 6 mo following HIV diagnosis stratified by HIV exposure group: COHERE 2000–2011. Male and females belonging to the “Other” HIV exposure group were combined into a single category. (DOCX) [file pmed.1001510.s005.docx]

| HIV |  |  | Univariate | | Multivariate | |
| --- | --- | --- | --- | --- | --- | --- |
| Exposure |  |  | OR (95% CI) | P | OR (95% CI) | P |
| Males | European | South | 1.00 | - | 1.00 | - |
| Having | Region | Central | 0.50 (0.45-0.56) | <0.0001 | 0.58 (0.52-0.64) | <0.0001 |
| Sex with | Of care | North | 0.36 (0.34-0.38) | <0.0001 | 0.39 (0.36-0.42) | <0.0001 |
| Males |  | East | 2.48 (2.03-3.04) | <0.0001 | 2.25 (1.83-2.78) | <0.0001 |
|  | Region of | Europe | 1.00 | - | 1.00 | - |
|  | Origin | Africa | 1.25 (0.99-1.59) | 0.064 | 0.95 (0.74-1.22) | 0.68 |
|  |  | Other | 1.03 (0.91-1.16) | 0.63 | 0.98 (0.87-1.11) | 0.75 |
|  |  | Unknown | 1.29 (1.22-1.37) | <0.0001 | 1.05 (0.99-1.12) | 0.14 |
|  | Age | /10 yrs older | 0.77 (0.74-0.79) | <0.0001 | 0.77 (0.75-0.80) | <0.0001 |
|  | HIV diagnosis | /yr later | 0.85 (0.84-0.85) | <0.0001 | 0.86 (0.85-0.87) | <0.0001 |
| Male | European | South | 1.00 | - | 1.00 | - |
| Heterosexual | Region | Central | 0.56 (0.48-0.66) | <0.0001 | 0.64 (0.54-0.76) | <0.0001 |
|  | Of care | North | 0.46 (0.42-0.51) | <0.0001 | 0.50 (0.45-0.55) | <0.0001 |
|  |  | East | 3.89 (3.12-4.84) | <0.0001 | 3.80 (2.99-4.83) | <0.0001 |
|  | Region of | Europe | 1.00 | - | 1.00 | - |
|  | Origin | Africa | 1.29 (1.16-1.44) | <0.0001 | 1.00 (0.89-1.13) | 0.96 |
|  |  | Other | 0.80 (0.66-0.96) | 0.015 | 0.74 (0.61-0.89) | 0.0017 |
|  |  | Unknown | 1.26 (1.15-1.38) | <0.0001 | 1.10 (0.99-1.23) | 0.074 |
|  | Age | /10 yrs older | 0.83 (0.80-0.87) | <0.0001 | 0.86 (0.83-0.89) | <0.0001 |
|  | HIV diagnosis | /yr later | 0.88 (0.87-0.89) | <0.0001 | 0.89 (0.88-0.91) | <0.0001 |
| Female | European | South | 1.00 | - | 1.00 | - |
| Heterosexual | Region | Central | 0.45 (0.39-0.53) | <0.0001 | 0.51 (0.43-0.61) | <0.0001 |
|  | Of care | North | 0.40 (0.36-0.43) | <0.0001 | 0.41 (0.38-0.45) | <0.0001 |
|  |  | East | 2.98 (2.53-3.50) | <0.0001 | 2.93 (2.42-3.55) | <0.0001 |
|  | Region of | Europe | 1.00 | - | 1.00 | - |
|  | Origin | Africa | 1.07 (0.97-1.17) | 0.19 | 0.92 (0.81-1.03) | 0.14 |
|  |  | Other | 0.75 (0.65-0.88) | 0.0002 | 0.80 (0.68-0.95) | 0.0092 |
|  |  | Unknown | 1.02 (0.93-1.12) | 0.64 | 1.10 (0.99-1.23) | 0.079 |
|  | Age | /10 yrs older | 0.83 (0.80-0.86) | <0.0001 | 0.88 (0.85-0.91) | <0.0001 |
|  | HIV diagnosis | /yr later | 0.87 (0.86-0.88) | <0.0001 | 0.89 (0.88-0.90) | <0.0001 |

| HIV |  |  | Univariate | | Multivariate | |
| --- | --- | --- | --- | --- | --- | --- |
| Exposure |  |  | OR (95% CI) | P | OR (95% CI) | P |
| Male | European | South | 1.00 | - | 1.00 | - |
| injecting | Region | Central | 0.55 (0.46-0.66) | <0.0001 | 0.55 (0.46-0.67) | <0.0001 |
| Drug | Of care | North | 0.21 (0.17-0.25) | <0.0001 | 0.20 (0.16-0.24) | <0.0001 |
| User |  | East | 3.77 (3.13-4.55) | <0.0001 | 2.63 (3.12-3.25) | <0.0001 |
|  | Region of | Europe | 1.00 | - | 1.00 | - |
|  | Origin | Africa | 0.75 (0.47-1.18) | 0.21 | 0.85 (0.53-1.39) | 0.52 |
|  |  | Other | 0.59 (0.37-0.94) | 0.027 | 0.66 (0.41-1.08) | 0.10 |
|  |  | Unknown | 0.73 (0.63-0.84) | <0.0001 | 0.70 (0.60-0.83) | <0.0001 |
|  | Age | /10 yrs older | 0.52 (0.48-0.57) | <0.0001 | 0.79 (0.72-0.87) | <0.0001 |
|  | HIV diagnosis | /yr later | 0.86 (0.84-0.88) | <0.0001 | 0.87 (0.85-0.89) | <0.0001 |
| Female | European | South | 1.00 | - | 1.00 | - |
| injecting | Region | Central | 1.02 (0.71-1.47) | 0.91 | 0.80 (0.55-1.17) | 0.25 |
| Drug | Of care | North | 0.39 (0.28-0.54) | <0.0001 | 0.33 (0.23-0.46) | <0.0001 |
| User |  | East | 6.60 (4.93-8.84) | <0.0001 | 4.33 (3.07-6.10) | <0.0001 |
|  | Region of | Europe | 1.00 | - | 1.00 | - |
|  | Origin | Africa | 0.18 (0.09-0.34) | <0.0001 | 0.25 (0.13-0.49) | <0.0001 |
|  |  | Other | 0.06 (0.01-0.46) | 0.0063 | 0.09 (0.01-0.66) | 0.018 |
|  |  | Unknown | 0.90 (0.70-1.15) | 0.33 | 1.39 (1.05-1.84) | 0.021 |
|  | Age | /10 yrs older | 0.47 (0.41-0.54) | <0.0001 | 0.71 (0.61-0.83) | <0.0001 |
|  | HIV diagnosis | /yr later | 0.84 (0.81-0.88) | <0.0001 | 0.87 (0.83-0.91) | <0.0001 |
| Other | European | South | 1.00 | - | 1.00 | - |
| (male/ | Region | Central | 0.55 (0.44-0.68) | <0.0001 | 0.88 (0.70-1.11) | 0.28 |
| female) | Of care | North | 1.21 (1.11-1.31) | <0.0001 | 1.64 (1.49-1.80) | <0.0001 |
|  |  | East | 1.75 (1.25-2.47) | 0.0012 | 3.56 (2.47-5.14) | <0.0001 |
|  | Region of | Europe | 1.00 | - | 1.00 | - |
|  | Origin | Africa | 1.60 (1.37-1.88) | <0.0001 | 1.69 (1.43-2.00) | <0.0001 |
|  |  | Other | 1.20 (0.96-1.49) | 0.11 | 1.24 (0.98-1.56) | 0.071 |
|  |  | Unknown | 2.86 (2.56-3.19) | <0.0001 | 3.04 (2.70-3.42) | <0.0001 |
|  | Age | /10 yrs older | 0.89 (0.88-0.92) | <0.0001 | 0.92 (0.89-0.96) | <0.0001 |
|  | HIV diagnosis | /yr later | 0.89 (0.88-0.90) | <0.0001 | 0.86 (0.85-0.88) | <0.0001 |
